# Role of Polar Phonons in the Photo Excited State of Metal Halide Perovskites

**DOI:** 10.1038/srep28618

**Published:** 2016-06-28

**Authors:** Menno Bokdam, Tobias Sander, Alessandro Stroppa, Silvia Picozzi, D. D. Sarma, Cesare Franchini, Georg Kresse

**Affiliations:** 1 University of Vienna, Faculty of Physics and Center for Computational Materials Science, Sensengasse 8/12, 1090 Vienna, Austria; 2 Consiglio Nazionale delle Ricerche - CNR-SPIN, I-67100 L’ Aquila, Italy; 3 Solid State and Structural Chemistry Unit, Indian Institute of Science, 560012 Bengaluru, India

## Abstract

The development of high efficiency perovskite solar cells has sparked a multitude of measurements on the optical properties of these materials. For the most studied methylammonium(MA)PbI_3_ perovskite, a large range (6–55 meV) of exciton binding energies has been reported by various experiments. The existence of excitons at room temperature is unclear. For the MAPb*X*_3_ perovskites we report on relativistic Bethe-Salpeter Equation calculations (*GW*-BSE). This method is capable to directly calculate excitonic properties from first-principles. At low temperatures it predicts exciton binding energies in agreement with the reported ‘large’ values. For MAPbI_3_, phonon modes present in this frequency range have a negligible contribution to the ionic screening. By calculating the polarization in time from finite temperature molecular dynamics, we show that at room temperature this does not change. We therefore exclude ionic screening as an explanation for the experimentally observed reduction of the exciton binding energy at room temperature and argue in favor of the formation of polarons.

In the last three years metal halide perovskites have come up as very promising solar cell materials^1^,^2^,^3^. Because of their relatively simple production procedure and high photovoltaic efficiency, they bear the potential of becoming competitive with current silicon based solar cells. The materials have an *OMX*_3_ perovskite structure (Organic(*O*), Metal(*M*), Halide(*X*)) and depending on the temperature up to three different crystal phases. The most frequently studied material is MAPbI_3_. At temperatures above 333 K, the lead and iodine atoms form a cubic perovskite structure enclosing a methylammonium (MA) molecule^4^. Combinations with the halogens Cl and Br can also be made and result in perovskite structures with different volumes and larger band gaps, not ideally suited for solar applications. According to early experimental measurements from the 1990s and 2000s, the MAPb*X*_3_ perovskites are semiconductors with optical band gaps (Δ_opt_) ranging between ~1.6–3.1 eV^5^,^6^,^7^,^8^. The optical gap is slightly lower than the fundamental electronic band gap (Δ), because of the electron-hole (e-h) interaction present in the excited system. With the emergence of very efficient perovskite solar cells, the mechanism behind the material’s good energy conversion rate has become a focus of research. In this regard, one important issue is the relatively large exciton binding energies (E_xb_) reported for these materials, 6–55 meV for MAPbI_3_^5^,^7^,^9^,^10^,^11^,^12^,^13^,^14^,^15^ and 76 meV^7^ for MAPbBr_3_. Intriguingly, many reported values are higher than *k*_B_*T*, which should make it difficult for electrons and holes to separate after excitation. It is then a mystery why these materials are so efficient in converting solar energy to power. As a solution to this puzzle, it has been proposed that ionic contributions from the PbI_3_ framework and the rotational freedom of MA molecules contribute to the screening properties, thereby reducing the exciton binding energy^16^,^17^,^18^,^19^. Alternative explanations invoke the formation of polarons, quasiparticles dressed by the ionic lattice that might lower the band gap^19^ below the excitonic onset. Furthermore, recent experiments indicate that temperature also plays a role. Y. Yamada *et al*.^9^ measured a reduction of E_*xb*_ from ~30 meV at 13 K to ~6 meV at 300 K, and likewise, A. Miyata *et al*.^10^ measured a value of 16 ± 2 meV in the low temperature orthorhombic phase, but only a few meV at room temperature. Whether ionic screening does or does not affect E_xb_ is under debate^20^. The large range of the reported E_xb_ values indicate the need for a theoretical description. In this work we report about first principles calculations on these ionic systems and address excitons in their interplay with polar phonons.

The theoretical modeling of metal halide perovskites is extremely challenging as it involves the treatment of several subtle, but important effects that are difficult to compute accurately. The first issue is the lattice structure to consider. Temperature dependent crystal structures have been determined^4^, but uncertainties in the orientation of the organic part prevent an unequivocal structural resolution. The structural characteristics have been the subject of numerous studies based on different (Local Density, Generalized Gradient, van der Waals) DFT approximations^21^,^22^,^23^,^24^,^25^,^26^. For the cubic phase of MAPbI_3_, most calculations predict lattice constants in good to excellent agreement with experiment. However, differences in the orientation of the molecule and the resulting deformation of the unit cell have been reported. We address this issue here (i) by performing global search for minimum energy structures, (ii) calculating excitons for various unit cells, (iii) and finite temperature simulations. Second, the presence of heavy elements requires to consider relativistic effects including spin-orbit coupling (SOC)^27^,^28^. Furthermore, for a quantitative description of the electronic structure, it is essential to calculate many-body quasiparticle energies e.g. in the framework of the *GW* approximation^27^,^29^,^30^,^31^. Finally, to evaluate E_xb_ and calculate accurate optical spectra, it is necessary to account for the e-h interaction. This can be done by the Bethe-Salpeter equation (BSE) following *GW* calculations^31^,^32^— a computationally exceedingly challenging endeavor if spin-orbit coupling is taken into account. It is therefore not astonishing that previous work has often given unsatisfactory results. Several quasiparticle *GW* calculations have been reported recently^27^,^29^,^30^,^31^, but a fully relativistic treatment including spin-orbit interaction was only performed in refs 27 and 33. Although, BSE calculations have been reported, these often neglect relativistic effects^31^,^32^ and report much too large binding energies. Even if relativistic effects are accounted for, the binding energies (E_xb_ = 0.153 eV) are at least a *factor 3* too large compared to any experimental values^33^. As we will show here, we can entirely resolve this issue when sampling the Brillouin zone with sufficient accuracy.

## Computational method

The first-principles calculations use a plane-wave basis and the projector augmented wave (PAW) method^34^ as implemented in the vasp code^35^,^36^,^37^. For structure determination, the PBEsol (Perdew, Burke, Ernzerhof modified for solids)^38^ functional was used, if not otherwise noted. Cross checks were also performed using van der Waals corrected functionals, specifically, the PBE-D3 method of Grimme^39^ finding no relevant differences for the properties reported here. The MAPb*X*_3_ cubic perovskite unit cells (12 atoms per cell) were constructed starting from the cubic-phase of MAPbI_3_ determined by X-ray diffraction^4^ and seeking the global energy minimum by simulated annealing. To determine candidate structures, molecular dynamics simulations were performed with a linear decrease of the temperature from 800 K to 500 K in 50000 steps of 1.5 fs. Approximately every ~1000 steps a snapshot was taken and fully relaxed. This process was repeated from the lowest energy structure yet found. A unique global minimum was found for all considered materials (See Supplementary Materials). In the subsequent electronic structure calculations (*GW* and BSE), SOC was fully included, and for Pb the 5*s*^2^5*p*^6^5*d*^10^ orbitals were included in the valence^27^. Gaussian smearing with *σ* = 0.05 eV was used to broaden the one-electron levels. Many-body effects were accounted for by first calculating PBE orbitals, and then determining the quasiparticle energies and fundamental gaps in the *GW*_0_ approximation^40^,^41^. Here the one electron energies in *G* were iterated until the quasiparticle energies are converged, while keeping *W*_0_ fixed at the DFT-RPA level^42^. About 2100 empty bands on a 4 × 4 × 4 Γ-centered k-point grid and 128 points on the frequency grid are needed to obtain well converged band gaps.

To determine the optical properties, the Bethe-Salpeter equation for the polarizability^43^,^44^,^45^ was solved. The common Tamm-Dancoff approximation^46^, 32 occupied and unoccupied KS orbitals, the *W*_0_ of the preceding *GW*_0_ calculations, and 6 × 6 × 6 k-points centered on a low symmetry k-point were used.

**Figure 1:**
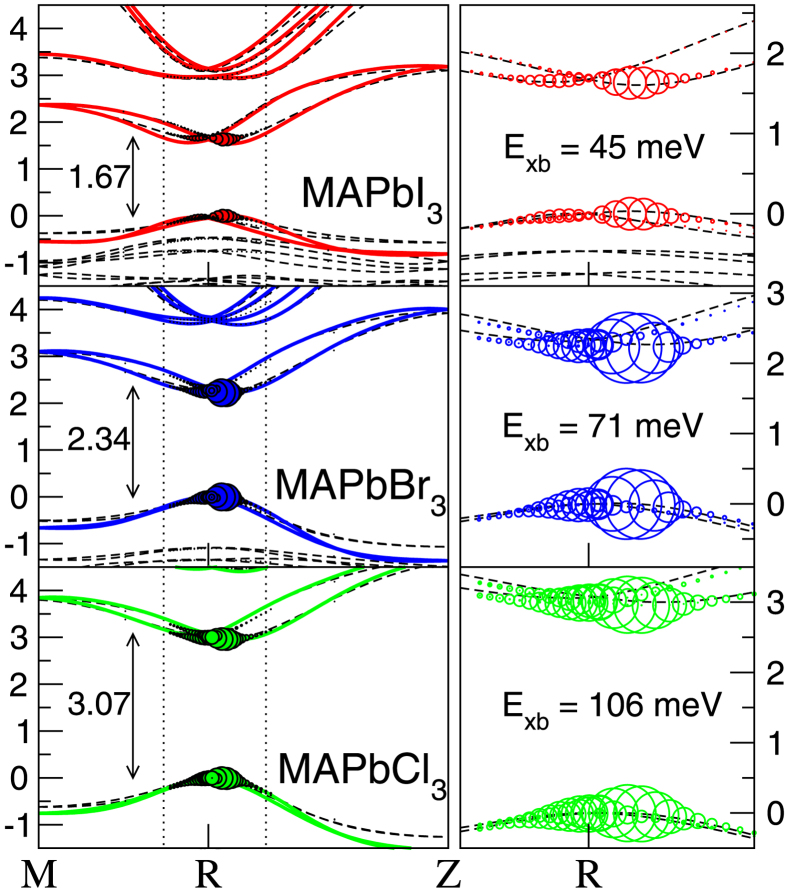
Fast band picture of excitons in MAPb*X*_3_. Left: *GW*_0_ band structure of MAPb*X*_3_ in the pseudo-cubic phase, with *X* = I (red), Br (blue) and Cl (green) determined by Wannier interpolation^64^ from a calculation using 4 × 4 × 4 k-points. The band gaps at *R* are indicated in eV. The dashed lines in the background are the corresponding DFT+scissor band structures. Right: Zoom-in of the band structure (marked by the dotted lines) close to the *R* point. The radii of the circles represent the contribution of the e-h pair at that k-point (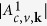) to the first exciton wave function.

To obtain k-point converged values for the exciton binding energy E_xb_ at least 20 × 20 × 20 k-points are, however, required. These BSE calculations, were performed using only 2 (un)occupied orbitals and fitting *W*_0_ to a model dielectric function^47^ that depends parametrically on the macroscopic dielectric constant determined in the previous BSE calculations with few k-points. Since even *GW* calculations are prohibitive for so many k-points, we use PBE calculations and applied a scissor technique to raise the unoccupied KS eigenvalues (compare Fig. 1). At these dense k-point grids, the E_xb_ becomes linearly dependent on the inverse of the total number of k-points^48^. The E_xb_ values reported in this work are therefore obtained by linear extrapolation to obtain the limit of the infinitely dense k-point grid (See Supplementary Materials).

The effect of different molecular orientations on the exciton binding energy have been assessed by BSE calculations on low energy configurations of the 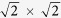 FASnI_3_ and MAPbI_3_ super cells. In addition, we have constructed a 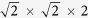 super cell for FASnI_3_. These structures were acquired by taking snap shots from Parallel Tempering Molecular Dynamics (PTMD) calculations at 300 K. The 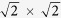 structures are the lowest energy configurations from the PTMD trajectory and were relaxed into their instantaneous ground state, while keeping the volume and cell shape fixed to the experiment. The 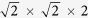 structure is a randomly picked configuration at 300 K from a separate PTMD calculation and was not relaxed. In the 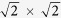 structures the molecular dipoles are orthogonally orientated w.r.t. each other and in the 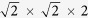 structures all the molecular dipoles have a different orientation. The same BSE calculation procedure was used as before, but the screening parameters and *GW*_0_ gap were not calculated; the values for the unit cell were used instead. This is a reasonable approximation, since calculation of the screening in the computationally more efficient random phase approximation shows little difference in electronic screening for different unit cells and different molecular orientations.

Details of the finite temperature dielectric function calculations are presented in the results section.

## Results

In Fig. 1 (left), the calculated *GW*_0_ quasiparticle band structures of the three MAPb*X*_3_ structures are shown. The band gap at the *R* points is indicated and is in excellent agreement with experiment^5^,^6^,^7^,^8^. SOC shifts the band gap minimum to *R′* making it slightly indirect^30^. The exciton wave function is expressed in an electron-hole product basis, 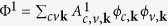. The first eigenstate 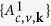 of the generalized BSE eigenvalue problem^45^ is visualized by plotting 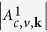 as a fat band structure. On the right hand side of Fig. 1 a zoom-in of the region close to *R* is made. It shows that the exciton is very localized in k-space, primarily consisting of states at the band extrema. Going from iodine to chlorine, the dispersion flattens (effective electron/hole masses increase), the band gap increases and, as a result, the extent of the exciton in k-space increases. The corresponding parameters are tabulated in Table 1. We have calculated the corresponding exciton binding energies also in the Wannier-Mott (WM) model for screened Coulomb interacting e-h pairs in parabolic bands: 
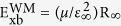
, with 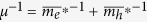 the effective mass of the e-h pair, *ε*_∞_ the high freq. dielectric constant and R_∞_ the Rydberg constant. Since we use the SOC split “Rashba-Dresselhaus”^49^ band structure in the BSE method, we can test the validity of the simple parabolic dispersion assumed in the WM model. We see (Table 1) that WM gives the correct order of the e-h interaction, however it results in a different ratio between I:Br and Br:Cl, which can not be trivially explained by small errors in *μ* or *ε*_∞_.

**Figure 2:**
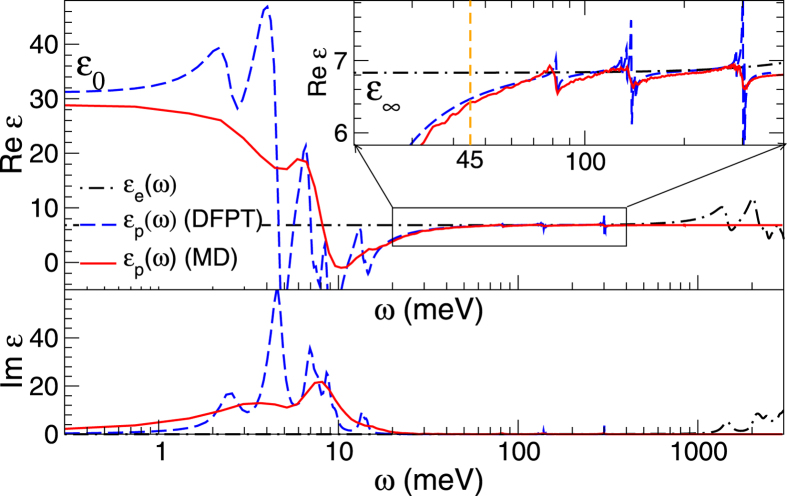
Ionic and electronic contributions (*ε*_p_, *ε*_e_) to the dielectric function *ε*(*ω*) of MAPbI^3^. The top/bottom figure shows the real/imaginary part of *ε*(*ω*). The solid red/dashed blue line are, respectively the results from the DFPT(T = 0 K)/MD(T = 300 K) method. The inset shows a zoom-in of R*eε*_p_(*ω*) close to the E_xb_ of 45 meV.

An important question is, whether the ionic contributions to the screening can be disregarded in the BSE calculations. To explore this point, Fig. 2 shows the sum of the ionic and electronic contribution to the dielectric function at 0 K, *ε*(*ω*), with the ionic contribution calculated using density functional perturbation theory (DFPT)^50^,^51^,^52^. A sizable increase of the static dielectric constant (*ε*_0_) compared to the ‘ion-clamped’ high frequency dielectric constant (*ε*_∞_) is found. The increase comes from optically active phonon modes below 20 meV (see Im *ε*_p_(*ω*)), clearly displaying the ionic nature of this material. However, the phonon modes present in the relevant energy window around E_xb_ ≈ 45 meV (see inset) are practically not active.

Since the exciton binding seems to change with temperature, the second intriguing question is whether the screening changes at finite temperature. To explore this, we have developed a novel scheme to evaluate the dielectric ionic response at finite temperature that we briefly describe in this section (a more detailed description will be presented in a future work). The idea is inspired by methods usually used to determine the *electronic contributions* to the screening in time-dependent DFT^53^. Well equilibrated finite temperature ensembles are subjected to a short constant electric field in time *Eδ*(*t*) acting on the *ions*. The *δ*-pulse is a natural way of exciting all possible frequencies in the system. The force exerted by this field onto the ions is proportional to 
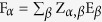
, where Z_*α*,*β*_ are the Born effective charges evaluated by density functional perturbation theory, and *α* and *β* are Cartesian indices^50^. In the first time step, these forces are added, thereby exciting the ionic system. The issue is to find a suitable way to calculate the induced ionic polarization P(*t*) caused by the delta peak. Here, we calculate the induced polarization as *δ*P(*t*) = (P_+_(*t*) − P_−_(*t*))/2, where P_+_(*t*) is the time evolution of the polarization for a positive delta peak *Eδ*(*t*), and P_−_(*t*) the time evolution of the polarization after a negative delta peak −*Eδ*(*t*). The evolving P(*t*) can in principle be evaluated using the Berry curvature^54^, but the Berry curvature often jumps discontinuously as the ions move. Hence, we evaluate the change of the polarization from the velocities *v*_*α*_(*t*) and the Born effective charges Z_*αβ*_(*t*) as 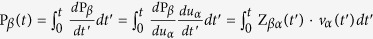. The additional cost is small, since Z(*t*) varies very slowly and needs to be recalculated only about every 50 time steps. The Fourier transformation of *δP*(*t*) is directly related to the ionic polarizability^55^. We first tested this approach at *T* = 0 K and found exact agreement with perturbation theory. To obtain reasonably noise-free data at finite temperature, we use a 2 × 2 × 2 super cell and average over 80 starting configurations in order to converge the spectrum. After the *δ*-pulse, the system is allowed to evolve in the micro-canonical ensemble unperturbed for 3 ps, the short time somewhat limiting the spectral resolution. However, the 80 starting configurations were obtained by a taking independent snapshots every 0.7 ps from a well equilibrated 60 ps long finite temperature MD trajectory. Therefore, we expect most of the dynamics to be sampled. The PBE-D3 method of Grimme^39^ was used here (although PBEsol results are very similar) and the deuterium mass was used for the hydrogen atoms. This replacement only changes the hydrogen related modes above 100 meV and allows to increase the time step during the simulation.

Clearly, the 300 K finite temperature polarizability (solid red line in Fig. 2) above 20 meV is very similar to the one at T = 0 K (dashed blue line in Fig. 2). The modes are at the same positions but broadened by fluctuations in the cage structure, as well as rotations of the molecules. Below 20 meV some differences are visible, however, in both methods the calculated *ε*_0_ is close to 30, in excellent agreement with the measured value of 28.8^56^.

We now discuss the question whether ionic screening should be included in the calculation of the exciton binding energy. The values reported in Table 1 assume a fixed lattice, i.e. a vertical transition. It is well established from the Franck-Condon energy diagram that lattice relaxation in the excited state can only *decrease* the transition energy, i.e. thermal or adiabatic transition energies including relaxation are always below vertical transition energies^57^. Lattice relaxation therefore can only increase the exciton binding energy, since the fundamental gap minus the transition energy is defined as the exciton binding energy E_xb_. Whether relaxation needs to be included, i.e. whether the vertical or thermal transition energy is measured experimentally can be disputed, although common wisdom is that optical absorption virtually always measures vertical excitation energies. One argument is that the position of the (dominant) longitudinal optical phonon mode (*ω*_L*O*_) compared to the typical energy scales of the optical absorption determines whether ionic relaxation should be considered. In the effective mass approximation this leads to two extreme cases, 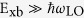, 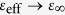 and 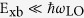 (ionic relaxation needs to be included)^58^,^59^. Since the dominant active phonon modes for MAPbI_3_ are all below 10 meV, we have 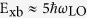 and the use of an *ε*_eff_ ≈ *ε*_∞_ ≈ 6.8 is entirely justified (see Fig. 2 for energies larger than 40 meV). The temperature independence of the ionic screening, furthermore, implies that the observed lowering of the exciton binding energy at elevated temperatures must have a different origin than changes in the ionic screening. In agreement with theory, recent room temperature time-resolved terahertz spectroscopy experiments, indicate a near constant screening *ε*(*ω*) = 5.5 in the frequency range of *ω* = 40–100 meV and E_xb_ = 49 ± 3 meV^60^.

However, if the exciton binding energy E_xb_ is not lowered by ionic screening, what mechanism then leads to carrier separation at higher temperatures? Our calculations also shed light on this. Individual electrons *e*^−^ and holes *h*^+^ can be screened by the lattice, thereby forming ‘dressed’ quasiparticles (QP) known as polarons. Since the mesoscopic Wannier-Mott model was so precise, we again resort to a mesosocopic model, namely, Fröhlich’s theory for large polarons. In this model, polaron formation lowers the QP energy by 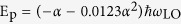, with a coupling constant 
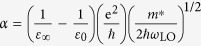
. Using the data from Table 1, a screening of *ε*_0_ = 30, *ε*_∞_ = 6, and 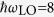 meV from Fig. 2 we obtain an *α* of 2.3/2.8. This lowers the QP energy of the electron and hole by 19 and 23 meV, respectively, and hence reduces the QP gap by 42 meV. This means that the charge separated polaronic state is only slightly less stable than the bound exciton. If we further recall that after excitation the electrons and holes are not yet close to the conduction or valence band edges, and that they are both individually scattered by lattice phonons loosing energy but possibly gaining momentum^61^, it is likely that they will rapidly separate in space and never reach their global groundstate, the bound exciton. Charge separation after optical excitation will be further eased by non-regularities in the electrostatic potential. And non-regularities exist aplenty in MAPbI_3_ at elevated temperatures: the polar MA molecules seem to prefer a short range ferroelectric order causing ferroelectric domains and a strong corrugation of the electrostatic potential^17^. A possible way to experimentally disentangle polaron formation and such molecular contributions and related corrugations in the potential is to perform control measurements on an *O*PbI_3_ perovskite with *O* cations that are non polar, for instance Cs.

**Figure 3:**
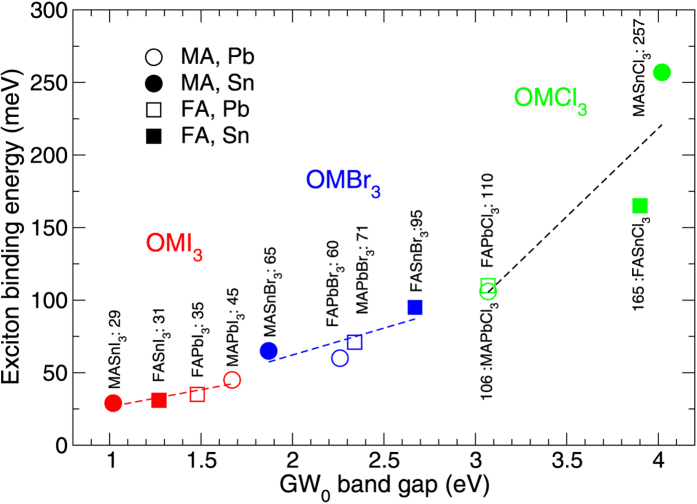
Calculated exciton binding energies and *GW*_0_ band gaps of twelve metal halide perovskites (*OMX*_3_, {*O* = MA, FA, *M* = Pb, Sn, X = I, Br, Cl}).

We like to comment briefly on the performance of a wider class of perovskites (*OMX*_3_). Specifically, we have replaced MA by formamidinium (FA) and Pb by Sn, thereby constructing twelve different perovskites. Their global lowest energy structure was calculated as before by simulated annealing and subsequent relaxation. FA is larger than MA and thereby changes the band gap of the perovskite. FASnI_3_ is a particular interesting candidate, previous work suggests that this is possibly a ferroelectric lead-free alternative for MAPbI_3_^49^. In Fig. 3 we show the trend in the exciton binding energies w.r.t. the *GW*_0_ band gap. Clearly, the halogen species predominantly determines the gap. For each halogen, the strength of the exciton binding energy and the optical gap can be fine-tuned by varying the molecule or the metal atom. Nevertheless, only iodine based perovskites seem to posses sufficiently small band gaps and exciton binding energies to be suitable for solar cells. An overview of the band gaps calculated at the various level of theory and available experimental data has been presented in Table 2. Over the whole range a good agreement is found between the *GW*-BSE calculations on these small unit cell structures and experimentally observed band gaps. Small discrepancies can be caused by the unit cell approach taken in this work. For those structures, which have not yet been synthesized or for which the band gap has not yet been measured, we put these number forward as predictions.

## Discussion

The last point we need to consider is how different molecular orientations influence the results. In a real (super)structure the exciton wave function will span many unit cells with molecules that have different orientations. It has been suggested that the ordering and orientation of the molecules in the lattice aids the e-h dissociation process^17^,^18^,^62^. Both the MA and FA molecules have an intrinsic dipole moment and are only weakly bonded to the *MX*_3_ cage. It is known from Nuclear Magnetic Resonance Spectroscopy measurements that the MA molecules in MAPb*X*_3_ have the full rotational degree of freedom at room temperature and that reorientation is a fairly rapid process^63^. However, with a typical reorientation time in the pico-second time scale, it is the slowest screening mechanism present in the *OMX*_3_ perovskites. To asses the effect of different molecular orientations, BSE calculations have been performed on 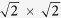 and 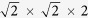 cells containing 2 and 4 molecules, respectively. For the 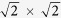 and the larger FASnI_3_ cell, the calculated exciton binding energies are 33 and 31 meV, respectively. Compared to the 31 meV predicted for the unit cell, the super cell approach does not give significantly different results for the exciton binding energy. The same holds for MAPbI_3_, where the 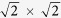 cell results in an exciton binding energy of 51 meV, which is only slightly larger than the 45 meV predicted for the unit cell.

## Conclusion

Accurate first principles calculations predict exciton binding energies of the order of 50, 70 and 110 meV for MAPbI_3_, MAPbBr_3_ and MAPbCl_3_, respectively. The agreement of the Wannier-Mott model with our high level calculations is good, provided that the model parameters are taken from accurate first principles calculations. The large exciton binding energy is clearly at variance with the observed high efficiency of solar cells, but in excellent agreement with most low temperature measurements. The much discussed ionic screening is almost temperature independent and substantially increases *ε*_0_ from around 6 to 30. However, the optically active modes are too slow (<10 meV) to effectively screen the excitons. For certain, we can rule out that a change of the ionic screening is responsible for the experimentally observed reduction of the exciton binding energy at room temperature. Instead, our calculations predict a different scenario: electrons and holes separate after optical excitation forming two individual polarons, lowering the fundamental gap by 42 meV. This scenario should now be carefully evaluated by experiments, and if validated, offers an intriguing option for the design of novel *polaronic* solar cell materials.

## Additional Information

**How to cite this article**: Bokdam, M. *et al*. Role of Polar Phonons in the Photo Excited State of Metal Halide Perovskites. *Sci. Rep.* **6**, 28618; doi: 10.1038/srep28618 (2016).

## Supplementary Material

Supplementary Information

## Figures and Tables

**Table 1:**
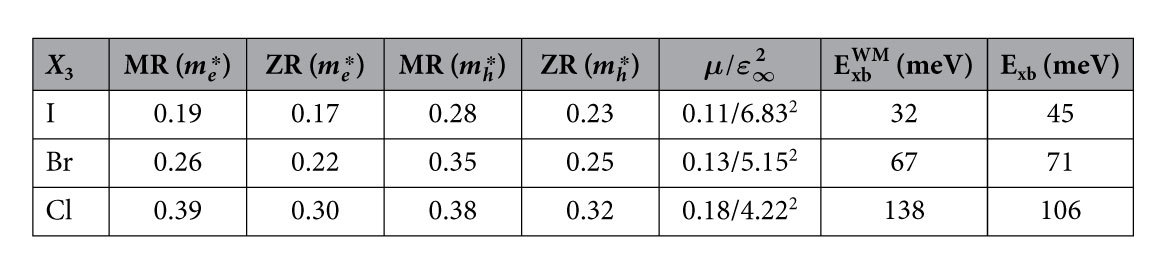
Effective electron and hole masses ( 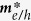 ) of the VBM and CBM in the MR and RZ directions from the *GW*_0_+ SOC band structures, the ratio of the exciton effective mass (*μ*) over the high freq. dielectric constant squared ( 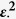 ), the Wannier-Mott -( 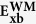 ) the BSE calculated (E_xb_) exciton binding energies.

**Table 2:**
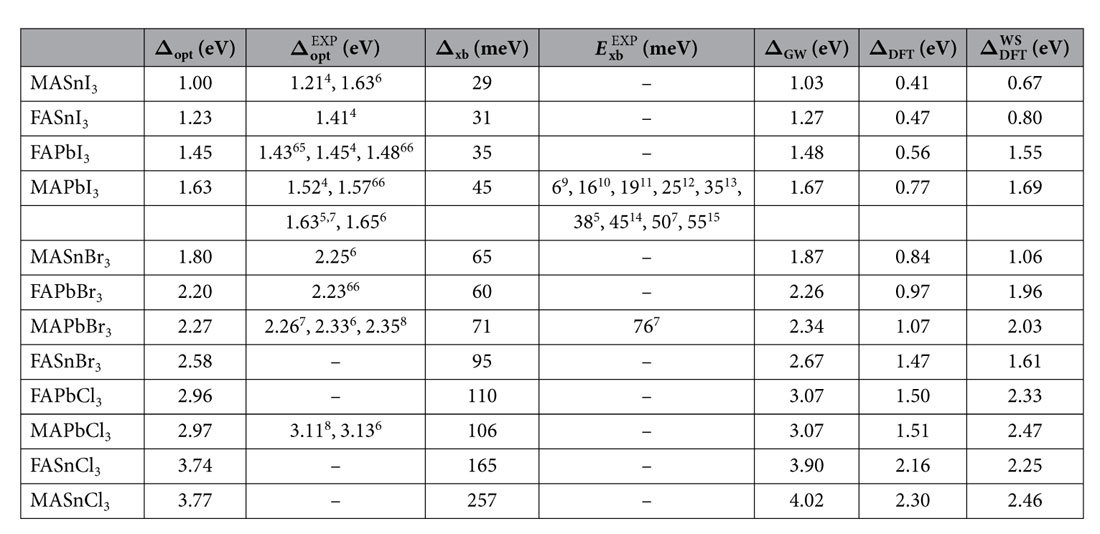
Calculated onset of optical absorption (Δ_opt_ = Δ_GW_ − E_xb_), exciton binding energy (E_xb_), the GW_0_ (Δ_GW_), DFT (Δ_DFT_) and DFT without SOC ( 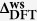) band gaps.

Available experimental results are shown for comparison.
