# Supplementary material for: Role of Polar Phonons in the Photo Excited State of Metal Halide Perovskites
Source: Sci Rep. 2016 Jun 28;6:28618. doi: 10.1038/srep28618 (PMC4923852; doi:10.1038/srep28618)
Supplement: Supplementary Information [file srep28618-s1.pdf]

Supplementary information to "Role of Polar Phonons in the Photo Excited State of Metal Halide Perovskites"

Menno Bokdam, Tobias Sander, Allesandro Stroppa,
Silvia Picozzi, D.D. Sarma, Cesare Franchini and Georg Kresse

Unit cell structures

The exciton binding energies presented in the paper have been calculated using 'semi-cubic' unit cells. These structures have been obtained by allowing all atoms to relax (keeping the experimental lattice shape and volume fixed) using damped relaxation algorithms with a $4 \times 4 \times 4$ k-point grid. The bromine and chlorine structures were constructed from the resulting high-symmetry iodine unit cells.

For these structures, in the first step all degrees of freedom were relaxed (including cell shape and volume) at a high energy cut off of 600 eV. Hereafter, a simulated annealing was performed, linearly cooling the structure from 800K to 500K in 50000 steps. Approximately every ∼1000 steps a snapshot was taken from the trajectory. For these structures the cell shape as well as the internal coordinates were relaxed and the lowest global energy structure was determined.

To confirm that the lowest energy structures for the primitive unit cells were correctly determined, a second molecular dynamics run was started from the first lowest energy structure. The same annealing procedure was used with the volume and cell shapes fixed to those of the yet best structures. More than 15 structures were picked from the MD and fully relaxed (including volume and cell shape). In only two cases, an even lower energy structure than in the first cycle was found, however, these two structures were only 15 meV lower in energy than in the first cycle. We are therefore confident that we have determined the lowest energy structures for the primitive unit cells reliably. Furthermore, all structures were carefully checked for instabilities in the vibrational frequencies and no instabilities were found, whereas virtually all initial structures constructed from experimental data alone exhibited such instabilities, even after careful relaxation. The resulting structures have been attached to this document.

Model dielectric function

A local model dielectric function (ε_{m}) [1] has been used in the BSE calculations to converge the exciton binding energies on dense k-point grids.

$$\varepsilon^{-1}(\mathbf{k}+\mathbf{G}) = 1 - (1-\varepsilon_{\mathrm{m}}^{-1})e^{\frac{-(2\pi|\mathbf{k}+\mathbf{G}|)^2}{4\lambda^2}}$$

A local function makes the screened Coulomb kernel diagonal ($\mathbf{G} = \mathbf{G}'$) in the screened Coulomb potential and allows us to calculate the high resolution plots of Fig. 1 (right) in the paper. The parameter ε_{m} comes from DFPT calculations on a shifted $8 \times 8 \times 8$ k-point grid and the screening length parameter (λ) was fitted to match the diagonal ($\mathbf{G} = \mathbf{G}'$) part of dielectric function from the GW_0 calculations on the shifted $6 \times 6 \times 6$ k-point grid, see Table 1. This approximation is also used in Ref. [2], however in that work a different functional was used. This approximation works very well, in particularly in the low energy part as is illustrated in Fig. 1. The imaginary part of the dielectric function calculated with the 'normal' GW-BSE matches with the one calculated with the model BSE (mBSE) method. Both calculations were performed using a $4 \times 4 \times 4$ k-point grid with the same KS orbital basis and GW_0 quasiparticle energies.

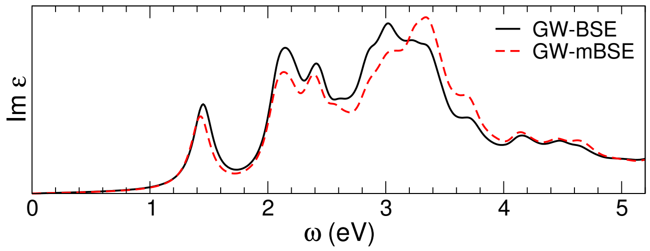

Figure 1: Imaginary part of $\varepsilon(\omega)$ calculated with the GW-BSE and GW-(model)BSE method. ($MAPbI_3$)

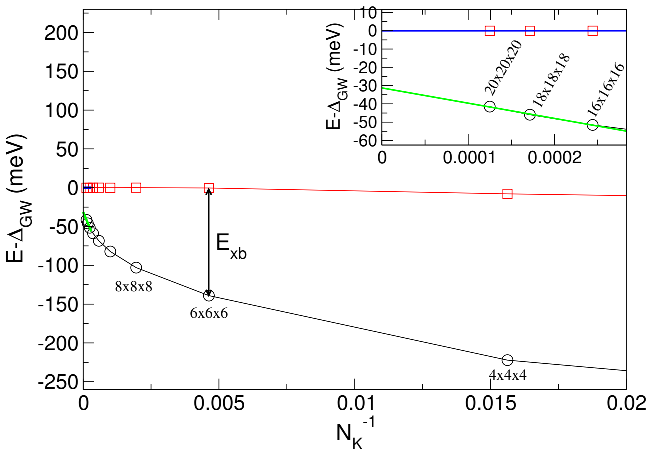

Figure 2: Eigenvalue of the first exciton with e-h interactions (circles) and in the independent particle picture (squares). The exciton binding energy is the difference between these two levels. The inset shows a zoom-in for the levels calculated at dense k-meshes. The green and blue lines are linear fits to the last three data points. (Example: $FASnI_3$)

Table 1: Parameters of the model dielctric function. Fitted screening length parameter (λ) and the "ion clamped" static dielectric constant (ε_∞) calculated using DFPT on a shifted $8 \times 8 \times 8$ k-point grid.

X_3	λ	ε_∞
$MASnI_3$	1.05	9.18
$FASnI_3$	1.05	8.06
$FAPbI_3$	1.05	7.10
$MAPbI_3$	1.05	6.83
$MASnBr_3$	1.13	5.89
$FAPbBr_3$	1.13	5.25
$MAPbBr_3$	1.13	5.15
$FASnBr_3$	1.13	5.32
$FAPbCl_3$	1.17	4.27
$MAPbCl_3$	1.17	4.22
$FASnCl_3$	1.17	4.07
$MASnCl_3$	1.17	4.05

K-point convergence

The calculated exciton binding energies have been carefully converged with respect to the k-point grid density. Coarse meshes result in exciton binding energies up to an order too large. The exciton binding energies presented in the paper are the result of a linear extrapolation to an infinite k-point grid[2] as is illustrated in Figure 2.

References

[1] Bechstedt, F., Sole, R. D., Cappellini, G. & Reining, L. An efficient method for calculating quasiparticle energies in semiconductors. *Sol. State Comm.* **84**, 765 – 770 (1992).

[2] Fuchs, F., Rödl, C., Schleife, A. & Bechstedt, F. Efficient $\mathcal{O}(N^2)$ approach to solve the bethe-salpeter equation for excitonic bound states. *Phys. Rev. B* **78**, 085103 (2008).

All unit cell structures have been added and formatted as Crystallographic Information File (CIF) files. The CIF formatted files can, for instance, be viewed with the freely available atomic structure visualization package VESTA (http://jp-minerals.org/vesta/).

```
%%%%%%%%%%%%%%%%%%%%%%%%%%%%%%%%%%%%%%%%%%%%%%%%%%%%%%%%%%%%%%%%%%%%%%%%%%%%%%%%
%%%%%%%%%%%%%%%%%%%%%%%%%%%%-------IODINES-------%%%%%%%%%%%%%%%%%%%%%%%%%%%%%%%%
%%%%%%%%%%%%%%%%%%%%%%%%%%%%%%%%%%%%%%%%%%%%%%%%%%%%%%%%%%%%%%%%%%%%%%%%%%%%%%%%

_pd_phase_name                         'MAPbI3'
_cell_length_a                         6.3115
_cell_length_b                         6.3115
_cell_length_c                         6.3161
_cell_angle_alpha                      90
_cell_angle_beta                       90
_cell_angle_gamma                      90
_symmetry_space_group_name_H-M         'P 1'
_symmetry_Int_Tables_number            1


loop_
   _atom_site_label
   _atom_site_occupancy
   _atom_site_fract_x
   _atom_site_fract_y
   _atom_site_fract_z
   _atom_site_adp_type
   _atom_site_B_iso_or_equiv
   _atom_site_type_symbol
   Pb1        1.0     0.4861      0.4861      0.4875     Biso  1.000000 Pb
   I1         1.0     0.5120      0.9835      0.4332     Biso  1.000000 I
   I2         1.0     0.9835      0.5120      0.4332     Biso  1.000000 I
   I3         1.0     0.4532      0.4532      0.9702     Biso  1.000000 I
   C1         1.0     0.9915      0.9915      0.9155     Biso  1.000000 C
   N1         1.0     0.0237      0.0237      0.1454     Biso  1.000000 N
   H1         1.0     0.9256      0.9256      0.2345     Biso  1.000000 H
   H2         1.0     0.1796      0.9924      0.1930     Biso  1.000000 H
   H3         1.0     0.9924      0.1796      0.1930     Biso  1.000000 H
   H4         1.0     0.0986      0.0986      0.8292     Biso  1.000000 H
   H5         1.0     0.0279      0.8258      0.8756     Biso  1.000000 H
   H6         1.0     0.8258      0.0279      0.8758     Biso  1.000000 H
```

```
%%%%%%%%%%%%%%%%%%%%%%%%%%%%%%%%%%%%%%%%%%%%%%%%%%%%%%%%%%%%%%%%%%%%%%%%%%%%%%%%%%
_pd_phase_name                          'FAPbI3'
_cell_length_a                          6.3521
_cell_length_b                          6.3521
_cell_length_c                          6.3521
_cell_angle_alpha                       89.9802
_cell_angle_beta                        89.9802
_cell_angle_gamma                       89.9802
_symmetry_space_group_name_H-M          'P 1'
_symmetry_Int_Tables_number             1

loop_
_symmetry_equiv_pos_as_xyz
   'x, y, z'

loop_
   _atom_site_label
   _atom_site_occupancy
   _atom_site_fract_x
   _atom_site_fract_y
   _atom_site_fract_z
   _atom_site_adp_type
   _atom_site_B_iso_or_equiv
   _atom_site_type_symbol
   Pb1        1.0     0.4738      0.4781      0.4738     Biso  1.000000 Pb
   I1         1.0     0.5200      0.9790      0.5200     Biso  1.000000 I
   I2         1.0     0.4615      0.4943      0.9772     Biso  1.000000 I
   I3         1.0     0.9772      0.4943      0.4615     Biso  1.000000 I
   N1         1.0     0.0504      0.0274      0.7929     Biso  1.000000 N
   N2         1.0     0.7929      0.0274      0.0504     Biso  1.000000 N
   H1         1.0     0.0303      0.8062      0.0303     Biso  1.000000 H
   H2         1.0     0.7173      0.1604      0.9959     Biso  1.000000 H
   H3         1.0     0.7307      0.9601      0.1837     Biso  1.000000 H
   H4         1.0     0.9959      0.1604      0.7173     Biso  1.000000 H
   H5         1.0     0.1837      0.9601      0.7307     Biso  1.000000 H
   C1         1.0     0.9606      0.9485      0.9606     Biso  1.000000 C
```

```
%%%%%%%%%%%%%%%%%%%%%%%%%%%%%%%%%%%%%%%%%%%%%%%%%%%%%%%%%%%%%%%%%%%%%%%%%%%%%%%%

_pd_phase_name                         'FASnI3'
_cell_length_a                         6.3292
_cell_length_b                         6.3292
_cell_length_c                         6.3290
_cell_angle_alpha                      90
_cell_angle_beta                       90
_cell_angle_gamma                      90.0582
_symmetry_space_group_name_H-M         'P 1'
_symmetry_Int_Tables_number            1

loop_
_symmetry_equiv_pos_as_xyz
   'x, y, z'

loop_
   _atom_site_label
   _atom_site_occupancy
   _atom_site_fract_x
   _atom_site_fract_y
   _atom_site_fract_z
   _atom_site_adp_type
   _atom_site_B_iso_or_equiv
   _atom_site_type_symbol
   Sn1        1.0     0.3658      0.3658      0.5000     Biso  1.000000 Sn
   I1         1.0     0.3458      0.8880      0.5000     Biso  1.000000 I
   I2         1.0     0.8880      0.3458      0.5000     Biso  1.000000 I
   I3         1.0     0.4175      0.4175      0.0000     Biso  1.000000 I
   N1         1.0     0.8438      0.8438      0.1829     Biso  1.000000 N
   N2         1.0     0.8438      0.8438      0.8171     Biso  1.000000 N
   H1         1.0     0.7296      0.7296      0.1966     Biso  1.000000 H
   H2         1.0     0.7296      0.7296      0.8034     Biso  1.000000 H
   H3         1.0     0.9081      0.9081      0.3163     Biso  1.000000 H
   H4         1.0     0.9081      0.9081      0.6837     Biso  1.000000 H
   H5         1.0     0.0347      0.0347      0.0000     Biso  1.000000 H
   C1         1.0     0.9119      0.9119      0.0000     Biso  1.000000 C
```

```
%%%%%%%%%%%%%%%%%%%%%%%%%%%%%%%%%%%%%%%%%%%%%%%%%%%%%%%%%%%%%%%%%%%%%%%%%%%%%%%%%

_pd_phase_name                        'MASnI3'
_cell_length_a                        6.2302
_cell_length_b                        6.2302
_cell_length_c                        6.2316
_cell_angle_alpha                     90
_cell_angle_beta                      90
_cell_angle_gamma                     90
_symmetry_space_group_name_H-M        'P 1'
_symmetry_Int_Tables_number           1

loop_
_symmetry_equiv_pos_as_xyz
   'x, y, z'

loop_
   _atom_site_label
   _atom_site_occupancy
   _atom_site_fract_x
   _atom_site_fract_y
   _atom_site_fract_z
   _atom_site_adp_type
   _atom_site_B_iso_or_equiv
   _atom_site_type_symbol
   Sn1        1.0     0.0121      1.0000      0.9084     Biso  1.000000 Sn
   I1         1.0     0.5166      1.0000      0.8552     Biso  1.000000 I
   I2         1.0     0.9760      1.5000      0.8634     Biso  1.000000 I
   I3         1.0     0.0583      1.0000      0.3812     Biso  1.000000 I
   C1         1.0     0.5313      0.5000      0.3335     Biso  1.000000 C
   N1         1.0     0.4546      1.5000      0.5583     Biso  1.000000 N
   H1         1.0     0.7078      0.5000      0.3332     Biso  1.000000 H
   H2         1.0     0.4708      0.3552      0.2526     Biso  1.000000 H
   H3         1.0     0.4708      0.6448      0.2526     Biso  1.000000 H
   H4         1.0     0.2876      1.5000      0.5711     Biso  1.000000 H
   H5         1.0     0.5072      1.3644      0.6422     Biso  1.000000 H
   H6         1.0     0.5072      1.6356      0.6422     Biso  1.000000 H
```

```
%%%%%%%%%%%%%%%%%%%%%%%%%%%%%%%%%%%%%%%%%%%%%%%%%%%%%%%%%%%%%%%%%%%%%%%%%%%%%%%%%
%%%%%%%%%%%%%%%%%%%%%%%%%%%%-------BROMINES-------%%%%%%%%%%%%%%%%%%%%%%%%%%%%%%%%
%%%%%%%%%%%%%%%%%%%%%%%%%%%%%%%%%%%%%%%%%%%%%%%%%%%%%%%%%%%%%%%%%%%%%%%%%%%%%%%%%

_pd_phase_name                        'FAPbBr3'
_cell_length_a                        5.9851
_cell_length_b                        5.9403
_cell_length_c                        5.9834
_cell_angle_alpha                     86.3525
_cell_angle_beta                      85.3027
_cell_angle_gamma                     85.4158
_symmetry_space_group_name_H-M        'P 1'
_symmetry_Int_Tables_number           1

loop_
_symmetry_equiv_pos_as_xyz
   'x, y, z'

loop_
   _atom_site_label
   _atom_site_occupancy
   _atom_site_fract_x
   _atom_site_fract_y
   _atom_site_fract_z
   _atom_site_adp_type
   _atom_site_B_iso_or_equiv
   _atom_site_type_symbol
   Pb1        1.0     0.3917      0.4790      0.4814     Biso  1.000000 Pb
   Br1        1.0     0.3400      0.9781      0.5293     Biso  1.000000 Br
   Br2        1.0     0.8885      0.4863      0.4462     Biso  1.000000 Br
   Br3        1.0     0.4203      0.4717      0.9842     Biso  1.000000 Br
   N1         1.0     0.8268      0.8355      0.8780     Biso  1.000000 N
   N2         1.0    -0.0000      0.1176      0.0324     Biso  1.000000 N
   H1         1.0     0.9416      0.8247      0.7416     Biso  1.000000 H
   H2         1.0     0.9944      0.2311      0.1562     Biso  1.000000 H
   H3         1.0     0.7004      0.7260      0.8940     Biso  1.000000 H
   H4         1.0     0.1294      0.1187      0.9089     Biso  1.000000 H
   H5         1.0     0.7090      0.9833      0.1721     Biso  1.000000 H
   C1         1.0     0.8405      0.9790      0.0327     Biso  1.000000 C
```

```
%%%%%%%%%%%%%%%%%%%%%%%%%%%%%%%%%%%%%%%%%%%%%%%%%%%%%%%%%%%%%%%%%%%%%%%%%%%%%%%%%

_pd_phase_name                         'FASnBr3'
_cell_length_a                         5.9933
_cell_length_b                         5.9612
_cell_length_c                         5.8864
_cell_angle_alpha                      88.0056
_cell_angle_beta                       92.2268
_cell_angle_gamma                      81.1984
_symmetry_space_group_name_H-M         'P 1'
_symmetry_Int_Tables_number            1

loop_
_symmetry_equiv_pos_as_xyz
   'x, y, z'

loop_
   _atom_site_label
   _atom_site_occupancy
   _atom_site_fract_x
   _atom_site_fract_y
   _atom_site_fract_z
   _atom_site_adp_type
   _atom_site_B_iso_or_equiv
   _atom_site_type_symbol
   Sn1        1.0     0.4478      0.3500      0.5365     Biso  1.000000 Sn
   Br1        1.0     0.4842      0.8866      0.5457     Biso  1.000000 Br
   Br2        1.0     0.9094      0.3337      0.5134     Biso  1.000000 Br
   Br3        1.0     0.4656      0.3372      0.0114     Biso  1.000000 Br
   N1         1.0     0.8300      0.7241      0.0364     Biso  1.000000 N
   N2         1.0     0.0654      0.9959      0.9873     Biso  1.000000 N
   H1         1.0     0.7319      0.7707      0.8896     Biso  1.000000 H
   H2         1.0     0.9884      0.0664      0.8363     Biso  1.000000 H
   H3         1.0     0.7915      0.5924      0.1363     Biso  1.000000 H
   H4         1.0     0.2004      0.0677      0.0470     Biso  1.000000 H
   H5         1.0     0.0913      0.7676      0.2609     Biso  1.000000 H
   C1         1.0     0.9986      0.8271      0.1001     Biso  1.000000 C
```

```
%%%%%%%%%%%%%%%%%%%%%%%%%%%%%%%%%%%%%%%%%%%%%%%%%%%%%%%%%%%%%%%%%%%%%%%%%%%%%%%%

_pd_phase_name                         'MAPbBr3'
_cell_length_a                         5.9083
_cell_length_b                         5.9079
_cell_length_c                         5.9638
_cell_angle_alpha                      93.8069
_cell_angle_beta                       93.7869
_cell_angle_gamma                      94.4654
_symmetry_space_group_name_H-M         'P 1'
_symmetry_Int_Tables_number            1

loop_
_symmetry_equiv_pos_as_xyz
   'x, y, z'

loop_
   _atom_site_label
   _atom_site_occupancy
   _atom_site_fract_x
   _atom_site_fract_y
   _atom_site_fract_z
   _atom_site_adp_type
   _atom_site_B_iso_or_equiv
   _atom_site_type_symbol
   Pb1        1.0     0.4981      0.5137      0.5544     Biso  1.000000 Pb
   Br1        1.0     0.4641      0.0140      0.5118     Biso  1.000000 Br
   Br2        1.0     0.9984      0.4798      0.5123     Biso  1.000000 Br
   Br3        1.0     0.5376      0.5524      0.0459     Biso  1.000000 Br
   C1         1.0     0.0294      0.0463      0.9767     Biso  1.000000 C
   N1         1.0     0.9579      0.9736      0.1959     Biso  1.000000 N
   H1         1.0     0.9022      0.1095      0.2922     Biso  1.000000 H
   H2         1.0     0.8271      0.8420      0.1734     Biso  1.000000 H
   H3         1.0     0.0945      0.9184      0.2917     Biso  1.000000 H
   H4         1.0     0.8830      0.1078      0.8824     Biso  1.000000 H
   H5         1.0     0.1666      0.1840      0.0069     Biso  1.000000 H
   H6         1.0     0.0912      0.9006      0.8815     Biso  1.000000 H
```

```
%%%%%%%%%%%%%%%%%%%%%%%%%%%%%%%%%%%%%%%%%%%%%%%%%%%%%%%%%%%%%%%%%%%%%%%%%%%%%%%%

_pd_phase_name                         'MASnBr3'
_cell_length_a                         5.8518
_cell_length_b                         5.8683
_cell_length_c                         5.9342
_cell_angle_alpha                      94.8877
_cell_angle_beta                       94.6696
_cell_angle_gamma                      94.72450
_symmetry_space_group_name_H-M         'P 1'
_symmetry_Int_Tables_number            1

loop_
_symmetry_equiv_pos_as_xyz
   'x, y, z'

loop_
   _atom_site_label
   _atom_site_occupancy
   _atom_site_fract_x
   _atom_site_fract_y
   _atom_site_fract_z
   _atom_site_adp_type
   _atom_site_B_iso_or_equiv
   _atom_site_type_symbol
   Sn1        1.0     0.9676      0.9780      0.8040     Biso  1.000000 Sn
   Br1        1.0     0.4484      0.0038      0.8444     Biso  1.000000 Br
   Br2        1.0     0.9929      0.4584      0.8549     Biso  1.000000 Br
   Br3        1.0     0.9287      0.9521      0.3312     Biso  1.000000 Br
   C1         1.0     0.4480      0.4372      0.3874     Biso  1.000000 C
   N1         1.0     0.5097      0.5220      0.1705     Biso  1.000000 N
   H1         1.0     0.3732      0.5740      0.4875     Biso  1.000000 H
   H2         1.0     0.3217      0.2863      0.3513     Biso  1.000000 H
   H3         1.0     0.6041      0.3893      0.4811     Biso  1.000000 H
   H4         1.0     0.6301      0.6661      0.1987     Biso  1.000000 H
   H5         1.0     0.5798      0.3959      0.0703     Biso  1.000000 H
   H6         1.0     0.3641      0.5638      0.0744     Biso  1.000000 H
```

```
%%%%%%%%%%%%%%%%%%%%%%%%%%%%%%%%%%%%%%%%%%%%%%%%%%%%%%%%%%%%%%%%%%%%%%%%%%%%%%%%%
%%%%%%%%%%%%%%%%%%%%%%%%%%%%-------CHLORINES-------%%%%%%%%%%%%%%%%%%%%%%%%%%%%%%
%%%%%%%%%%%%%%%%%%%%%%%%%%%%%%%%%%%%%%%%%%%%%%%%%%%%%%%%%%%%%%%%%%%%%%%%%%%%%%%%%

_pd_phase_name                        'FAPbCl3'
_cell_length_a                        5.7210
_cell_length_b                        5.7212
_cell_length_c                        5.7487
_cell_angle_alpha                     89.8621
_cell_angle_beta                      90.1348
_cell_angle_gamma                     81.6927
_symmetry_space_group_name_H-M        'P 1'
_symmetry_Int_Tables_number           1

loop_
_symmetry_equiv_pos_as_xyz
   'x, y, z'

loop_
   _atom_site_label
   _atom_site_occupancy
   _atom_site_fract_x
   _atom_site_fract_y
   _atom_site_fract_z
   _atom_site_adp_type
   _atom_site_B_iso_or_equiv
   _atom_site_type_symbol
   Pb1        1.0     0.4260      0.4361      0.4908     Biso  1.000000 Pb
   Cl1        1.0     0.3950      0.9283      0.4726     Biso  1.000000 Cl
   Cl2        1.0     0.9337      0.4670      0.4726     Biso  1.000000 Cl
   Cl3        1.0     0.4226      0.4394      0.0052     Biso  1.000000 Cl
   N1         1.0     0.0804      0.0493      0.9393     Biso  1.000000 N
   N2         1.0     0.8128      0.7816      0.9393     Biso  1.000000 N
   H1         1.0     0.1547      0.9974      0.7806     Biso  1.000000 H
   H2         1.0     0.8646      0.7073      0.7806     Biso  1.000000 H
   H3         1.0     0.1531      0.1789      0.0243     Biso  1.000000 H
   H4         1.0     0.6832      0.7089      0.0243     Biso  1.000000 H
   H5         1.0     0.8497      0.0124      0.2073     Biso  1.000000 H
   C1         1.0     0.9120      0.9500      0.0350     Biso  1.000000 C
```

```
%%%%%%%%%%%%%%%%%%%%%%%%%%%%%%%%%%%%%%%%%%%%%%%%%%%%%%%%%%%%%%%%%%%%%%%%%%%%%%%%

_pd_phase_name                         'FASnCl3'
_cell_length_a                         5.6714
_cell_length_b                         5.6710
_cell_length_c                         5.9083
_cell_angle_alpha                      91.0972
_cell_angle_beta                       88.9182
_cell_angle_gamma                      80.2096
_symmetry_space_group_name_H-M         'P 1'
_symmetry_Int_Tables_number            1

loop_
_symmetry_equiv_pos_as_xyz
   'x, y, z'

loop_
   _atom_site_label
   _atom_site_occupancy
   _atom_site_fract_x
   _atom_site_fract_y
   _atom_site_fract_z
   _atom_site_adp_type
   _atom_site_B_iso_or_equiv
   _atom_site_type_symbol
   Sn1        1.0     0.4509      0.3724      0.5605     Biso  1.000000 Sn
   Cl1        1.0     0.4952      0.9087      0.5428     Biso  1.000000 Cl
   Cl2        1.0     0.9145      0.3283      0.5424     Biso  1.000000 Cl
   Cl3        1.0     0.4375      0.3857      0.1280     Biso  1.000000 Cl
   N1         1.0     0.0522      0.0385      0.0058     Biso  1.000000 N
   N2         1.0     0.7851      0.7710      0.0064     Biso  1.000000 N
   H1         1.0     0.0063      0.1047      0.8480     Biso  1.000000 H
   H2         1.0     0.7185      0.8166      0.8486     Biso  1.000000 H
   H3         1.0     0.1736      0.1186      0.0925     Biso  1.000000 H
   H4         1.0     0.7053      0.6497      0.0935     Biso  1.000000 H
   H5         1.0     0.0000      0.8240      0.2738     Biso  1.000000 H
   C1         1.0     0.9477      0.8759      0.1018     Biso  1.000000 C
```

```
%%%%%%%%%%%%%%%%%%%%%%%%%%%%%%%%%%%%%%%%%%%%%%%%%%%%%%%%%%%%%%%%%%%%%%%%%%%%%%%%%%%

_pd_phase_name                         'MAPbCl3'
_cell_length_a                         5.6529
_cell_length_b                         5.6525
_cell_length_c                         5.7153
_cell_angle_alpha                      94.2224
_cell_angle_beta                       94.3444
_cell_angle_gamma                      94.9603
_symmetry_space_group_name_H-M         'P 1'
_symmetry_Int_Tables_number            1

loop_
_symmetry_equiv_pos_as_xyz
   'x, y, z'

loop_
   _atom_site_label
   _atom_site_occupancy
   _atom_site_fract_x
   _atom_site_fract_y
   _atom_site_fract_z
   _atom_site_adp_type
   _atom_site_B_iso_or_equiv
   _atom_site_type_symbol
   Pb1        1.0     0.5413      0.6291      0.5590     Biso  1.000000 Pb
   Cl1        1.0     0.5766      0.1283      0.6028     Biso  1.000000 Cl
   Cl2        1.0     0.0404      0.6642      0.6041     Biso  1.000000 Cl
   Cl3        1.0     0.5055      0.5917      0.0633     Biso  1.000000 Cl
   C1         1.0     0.0099      0.1016      0.1473     Biso  1.000000 C
   N1         1.0     0.0837      0.1707      0.9172     Biso  1.000000 N
   H1         1.0     0.2230      0.3076      0.9396     Biso  1.000000 H
   H2         1.0     0.1375      0.0250      0.8188     Biso  1.000000 H
   H3         1.0     0.9406      0.2267      0.8162     Biso  1.000000 H
   H4         1.0     0.1627      0.0388      0.2476     Biso  1.000000 H
   H5         1.0     0.9487      0.2571      0.2441     Biso  1.000000 H
   H6         1.0     0.8641      0.9581      0.1181     Biso  1.000000 H
```

```
%%%%%%%%%%%%%%%%%%%%%%%%%%%%%%%%%%%%%%%%%%%%%%%%%%%%%%%%%%%%%%%%%%%%%%%%%%%%%%%%%%

_pd_phase_name                         'MASnCl3'
_cell_length_a                         5.6339
_cell_length_b                         5.8254
_cell_length_c                         5.6358
_cell_angle_alpha                      84.9543
_cell_angle_beta                       83.0910
_cell_angle_gamma                      95.1251
_symmetry_space_group_name_H-M         'P 1'
_symmetry_Int_Tables_number            1

loop_
_symmetry_equiv_pos_as_xyz
   'x, y, z'

loop_
   _atom_site_label
   _atom_site_occupancy
   _atom_site_fract_x
   _atom_site_fract_y
   _atom_site_fract_z
   _atom_site_adp_type
   _atom_site_B_iso_or_equiv
   _atom_site_type_symbol
   Sn1        1.0     0.9913      0.0389      0.9481     Biso  1.000000 Sn
   Cl1        1.0     0.5247      0.9930      0.9834     Biso  1.000000 Cl
   Cl2        1.0     0.0032      0.5966      0.9361     Biso  1.000000 Cl
   Cl3        1.0     0.9559      0.9933      0.4147     Biso  1.000000 Cl
   C1         1.0     0.4971      0.4501      0.4432     Biso  1.000000 C
   N1         1.0     0.4394      0.6837      0.4998     Biso  1.000000 N
   H1         1.0     0.3370      0.3553      0.3909     Biso  1.000000 H
   H2         1.0     0.6448      0.4679      0.2942     Biso  1.000000 H
   H3         1.0     0.5518      0.3574      0.6030     Biso  1.000000 H
   H4         1.0     0.5894      0.7797      0.5456     Biso  1.000000 H
   H5         1.0     0.3920      0.7782      0.3499     Biso  1.000000 H
   H6         1.0     0.2985      0.6738      0.6413     Biso  1.000000 H

%%%%%%%%%%%%%%%%%%%%%%%%%%%%%%%%%%%%%%%%%%%%%%%%%%%%%%%%%%%%%%%%%%%%%%%%%%%%%%%%%%
```
